# New Delhi Metallo-β-Lactamase, Ontario, Canada

**DOI:** 10.3201/eid1702.101561

**Published:** 2011-02

**Authors:** Nathalie Tijet, David C. Alexander, David Richardson, Olga Lastovetska, Donald E. Low, Samir N. Patel, Roberto G. Melano

**Affiliations:** Author affiliations: Ontario Agency for Health Protection and Promotion, Toronto, Ontario, Canada (N. Tijet, D.C. Alexander, O. Lastovetska, D.E. Low, S.N. Patel, R.G. Melano);; University of Toronto, Toronto (D.C. Alexander, O. Lastovetska, D.E. Low, S.N. Patel);; William Osler Health Centre, Brampton Civic Hospital, Brampton, Ontario, Canada (D. Richardson);; Mount Sinai Hospital, Toronto (D.E. Low, R.G. Melano)

**Keywords:** Bacteria, Klebsiella pneumoniae, New Delhi metallo-β-lactamase, NDM-1, metallo-carbapenemase, multidrug resistance, horizontal transfer, Canada, expedited, letter

**To the Editor**: The New Delhi metallo-β-lactamase (NDM-1) was first characterized in 2009 from *Klebsiella pneumoniae* and *Escherichia coli* isolated from a patient in Sweden who had received medical care in New Delhi, India (*1*). Further studies have shown broad dissemination of this β-lactamase gene (*bla*_NDM-1_) in India, Pakistan, Bangladesh, and the United Kingdom (*2*). Additional isolates have been detected in other countries, and many of the patients with NDM-1–producing *Enterobacteriaceae* reported receiving medical care in the Indian subcontinent (*1**–**7*). We describe detection and characterization of an NDM-1–producing *K. pneumoniae* isolated in Ontario, Canada.

In August 2010, a urinary tract infection was diagnosed in a 36-year-old woman in a hospital in Brampton, Ontario. An *E. coli* strain sensitive to multiple antibacterial drugs (including carbapenems) was isolated from a midstream urine sample; the patient was successfully treated with ciprofloxacin. One week after treatment, when the patient did not have a fever or other clinical signs, a urine culture was repeated, and a carbapenem-resistant *K. pneumoniae* isolate (GN529) was recovered. Travel history indicated that the patient had recently returned from India, where in mid-July she had had a miscarriage and had been hospitalized in Mumbai for 2 days. At that time, no antimicrobial drug treatment was prescribed.

Susceptibility profiles of *K. pneumoniae* GN529 and its *E. coli* transconjugant were obtained by using Etest (bioMérieux, Marcy l’Etoile, France) and the agar dilution method based on the Clinical and Laboratory Standards Institute guidelines (*8*). Multilocus sequence typing (MLST) of isolate GN529 was performed as described (*9*). The Pasteur Institute online database (www.pasteur.fr/recherche/genopole/PF8/mlst/Kpneumoniae.html) was used to assign the allelic numbers and sequence type (ST).

To screen for the most commonly known β-lactamase genes in enterobacteria, we performed multiplex PCRs (*10*). Primers were designed (NDM-F, 5′-AATGGAATTGCCCAATATTATGC-3′; NDM-R, 5′-CGAAAGTCAGGCTGTGTTG C-3′) for the specific detection of *bla*_NDM-1_ and included in 1 of the multiplex PCRs (multiplex V). Primers NDM-F and NDM-R2 (5′-TCAGCGCAGCTTGTCGGC-3′) were used to amplify and sequence the entire *bla*_NDM-1_ gene. The samples were screened for the presence of six 16S methylase genes (*armA*, *rmtA–D*, and *npmA*) by PCR. *E. coli* J53 transconjugants were selected on Luria-Bertani plates containing sodium azide and meropenem (100 µg/mL and 1 µg/mL, respectively). The plasmid harboring *bla*_NDM-1_ was identified by Southern blot analysis by using a specific digoxigenin-labeled *bla*_NDM-1_ probe (Roche Diagnostics, Indianapolis, IN, USA).

*K. pneumoniae* GN529 was highly resistant to all β-lactams, aminoglycosides, quinolones, tetracycline, nitrofurantoin, and co-trimoxazole. MICs of 0.5 µg/mL for colistin (European Committee on Antimicrobial Susceptibility Testing colistin breakpoint for *Enterobacteriaceae*: susceptibility <2 μg/mL) and 1 μg/mL for tigecycline (European Committee on Antimicrobial Susceptibility Testing and US Food and Drug Administration tigecycline breakpoint for *Enterobacteriaceae*: susceptibility <1 and <2 μg/mL, respectively) were also obtained (Table).

**Table:** Antibacterial drug susceptibility profiles and resistance genes of *Klebsiella pneumoniae* GN529 clinical isolate and its *Escherichia coli* transconjugant, Ontario, Canada, 2010*

Antibacterial drug or gene	MIC, μg/mL		
	Kpn GN529	Eco J529	Eco J53
Ampicillin	>256	>256	6
Cefoxitin	>256	>256	8
Ceftazidime	>256	>256	0.19
Cefotaxime	>256	>256	0.094
Cefepime	48	48	0.064
Ertapenem	32	12	0.008
Meropenem	>32	4	0.023
Imipenem	>32	32	0.38
Amikacin	>256	>256	1.5
Gentamicin	>256	>256	1.5
Tobramycin	>256	>256	1
Ciprofloxacin	>32	0.012	0.012
Tetracycline	>16	0.78	1
Tigecycline	1	ND	ND
Nitrofurantoin	>512	ND	ND
Colistin	0.5	ND	ND
Co-trimoxazole	4/76	ND	ND
PCR† and sequencing			
*bla*_NDM-1_	+	+	ND
*bla*_CTX-M-15_	+	−	ND
*bla*_SHV-12_	+	+	ND
*bla*_SHV-11_	+	–	ND
*bla*_OXA-1_	+	–	ND
*bla*_TEM-1_	+	–	ND
* armA*	+	+	ND

*Kpn, *Klebsiella pneumoniae*; Eco J529, *Escherichia coli* transconjugant strain; Eco J53, recipient *E. coli* J53; ND, not determined. †PCR screening included *bla*_TEM_, *bla*_SHV_, *bla*_OXA-1_-like, *bla*_CTX-M_ groups 1, 2, and 9, *bla*_VEB_, *bla*_PER_, *bla*_GES_, *bla*_OXA-48_-like, *bla*_IMP_, *bla*_VIM_, *bla*_KPC_, *bla*_NDM-1_, and 6 groups of *bla*_AmpC_ genes.

Considering the travel history of the patient and the high level resistance to all β-lactams, molecular screening of β-lactamases in strain GN529 was initiated to identify possible carbapenemases (e.g., *bla*_NDM-1_) in that isolate. Five β-lactamases genes (*bla*_NDM_, *bla*_SHV_, *bla*_TEM_, group 1 *bla*_CTX-M_ , and *bla*_OXA_) and one 16S rRNA methylase (*armA*) were detected. By using primers for amplification of complete genes, we obtained sequences of *bla*_NDM-1_, 2 extended-spectrum β-lactamases (*bla*_CTX-M-15_ and *bla*_SHV-12_), 3 broad-spectrum β-lactamases (*bla*_SHV-11_, *bla*_TEM-1_ and *bla*_OXA-1_), and methyltransferase *armA*. No AmpC β-lactamases were linked to this isolate. Southern blotting identified a plasmid of ≈150 kb harboring *bla*_NDM-1_ (data not shown). A transconjugant *E. coli* positive for *bla*_NDM-1_ (*E. coli* J529, Table) was resistant to all β-lactams and aminoglycosides tested. In addition, *bla*_SHV-12_ and *armA* were detected in strain J529 (Table), indicating the potential for the horizontal spread of these resistance genes.

*K. pneumoniae* GN529 was typed by MLST as ST147, the same type as a clinical NDM-1–producing strain isolated in Australia (*6*) but distinct from ST14 and ST16 strains described (*1**,**7*). There are insufficient MLST data to confirm polyclonal dissemination of NDM-1, but previous pulsed-field gel electrophoresis results support that hypothesis (*2*).

*K. pneumoniae* GN529 was isolated from a patient who had recently received emergency medical care in India, suggesting importation of this clinical strain. In the United Kingdom, where *Enterobacteriaceae* containing *bla*_NDM-1_ are increasingly common, carriage of these organisms has been closely linked to receipt of medical care in the Indian subcontinent (*2*). Similar association as a risk factor was observed in other regions, including *bla*_NDM-1_-positive clinical strains isolated in North America, Australia, and Africa (*3**–**6**,**10*).

The NDM-1–producing enterobacteria described in this study previously had low MICs only for colistin and tigecycline (*1**,**2**,**5**,**6*). However, an NDM-1 isolate resistant to these antimicrobial drugs has also been described (*2*). Early detection and implementation of infection control interventions is essential for preventing the spread of multidrug-resistant organisms such as these. It may be prudent to consider medical exposure in the Indian subcontinent as a risk factor for possible infection, colonization, or both with multidrug-resistant, NDM-1–producing *Enterobacteriaceae*.
